# Fatigue in children and adolescents perinatally infected with human immunodeficiency virus: an observational study

**DOI:** 10.1186/s12887-021-02977-6

**Published:** 2021-11-20

**Authors:** A. M. ter Haar, M. M. Nap-van der Vlist, M. Van den Hof, S. L. Nijhof, R. R. L. van Litsenburg, K. J. Oostrom, D. Pajkrt

**Affiliations:** 1 Pediatric Infectious Diseases, Emma Children’s Hospital, Amsterdam University Medical Centers, Location Academic Medical Center, University of Amsterdam, Meibergdreef 9, 1105 AZ Amsterdam, the Netherlands; 2 Department of Paediatrics, Wilhelmina Children’s Hospital, University Medical Center Utrecht, University of Utrecht, Utrecht, The Netherlands; 3 Department of Paediatric Oncology, Princess Máxima Center for Paediatric Oncology, Utrecht, The Netherlands; 4 Psychosocial Department, Emma Children’s Hospital, Amsterdam University Medical Centers, Location Academic Medical Center, University of Amsterdam, Amsterdam, The Netherlands

**Keywords:** Perinatal HIV, Vertical HIV, HIV-infection, Pediatric, Child, Adolescent, Fatigue, Quality of life, Health-related quality of life

## Abstract

**Background:**

Fatigue is common among adults living with human immunodeficiency virus (HIV) as well as children with a chronic disease (CCD). Fatigue can have disastrous effects on health status, including health related quality of life (HRQOL). Even so, fatigue is underexplored in children and adolescents perinatally infected with HIV (PHIV+) in the Netherlands. The objective of this observational study is to explore fatigue in PHIV+ and its association with their HRQOL.

**Methods:**

We measured HRQOL and fatigue using the Pediatric Quality of Life Inventory™ (PedsQL 4.0) and the PedsQL Multidimensional Fatigue Scale (MFS). The PedsQL MFS encompasses three subscales: general fatigue, sleep/rest fatigue and cognitive fatigue, and a total fatigue score. We compared outcomes of PHIV+ children and adolescents in the Amsterdam University Medical Centre with three groups: 1) HIV-uninfected controls (HIV-) matched for age, sex, region of birth, socioeconomic status and adoption status, 2) CCD, and 3) the general Dutch population. Within the PHIV+ group we explored associations between fatigue and HRQOL.

**Results:**

We enrolled 14 PHIV+ (median age 10.2 years [IQR 9.2–11.4]) and 14 HIV-. Compared to CCD, PHIV+ significantly reported less general fatigue (mean difference 13.0, 95% CI 1.3 to 24.8). PHIV+ did not score significantly different on any of the other PedsQL MFS scales compared to HIV-, CCD or the general Dutch population. PHIV children scored relatively low on the cognitive fatigue scale in comparison to HIV-uninfected matched controls, CCD and the general population, although these differences did not reach significance. Among PHIV+, a lower score on total fatigue, general fatigue and cognitive fatigue was associated with a lower HRQOL score.

**Conclusions:**

The results of this study suggest that PHIV children and adolescents do not experience more symptoms of fatigue than their healthy peers. However, PHIV children and adolescents may be more likely to experience cognitive fatigue. Fatigue in PHIV also appears to be associated with children’s HRQOL. Further research should confirm these exploratory findings.

**Supplementary Information:**

The online version contains supplementary material available at 10.1186/s12887-021-02977-6.

## Background

Children and adolescents perinatally infected with HIV (PHIV+) are provided with advanced supportive healthcare in the Netherlands. This results in generally favorable outcomes, with a low mortality rate and good long-term virological and immunological responses to treatment [1]. Still, undesirable outcomes remain even in optimally treated and clinically stable PHIV+, including cognitive problems, lower IQ, and lower health related quality of life (HRQOL) [2–4]. Since PHIV+ can now be considered a chronic condition, there is a need to carefully monitor symptoms that are common amongst children living with a chronic disease (CCD) [5–8]. Fatigue is one of these commonly reported symptoms in CCD [9]. Likewise, fatigue is a common and disabling symptom among adult people living with HIV (PLWH) [10, 11]. However, we know little about symptoms of fatigue in children and adolescents with PHIV+.

Fatigue is a multidimensional construct, encompassing cognitive, behavioral, emotional and physiological aspects [12]. In children and adolescents with chronic disease, fatigue does not appear to be a disease-specific process [9] and can persist despite low disease activity [13]. Similarly, the etiology of HIV-related fatigue appears to be multifactorial [11, 14]. Important factors in PLWH with well controlled disease include an ongoing chronic inflammatory response [15, 16], disturbed sleep patterns [17], effects of long term combination antiretroviral therapy (cART) [15], and psychological factors including cognition [18], which is found to be poorer in PHIV+ [3, 4].

Experiencing fatigue on a daily basis can have a detrimental effect on concentration, focus and emotional well-being [19, 20]. Subsequently, this can lead to school absence, poor academic achievement and restrictions in social interactions with peers [21, 22]. Among adult PLWH, fatigue has been found to affect adherence to ART, possibly due to its disruptive impact on daily routines and on concentration and memory [23–25]. Fatigue can lead to adverse health outcomes and a lower health-related quality of life (HRQOL), and is thus an important and disabling symptom [26].

Studies have shown a high prevalence of fatigue in children with chronic disease as well as adult PLWH [9, 10], yet it has only been systematically studied in one adolescent group of PLWH in South Africa [19, 27, 28]. Within this group, about a quarter to half of the adolescents living with HIV demonstrated problematic levels of fatigue [27]. Since children or adolescents who acquired HIV perinatally have been living with the disease and using cART from an early age, they are likely to face different challenges and have different health needs compared with young adults with behaviorally acquired HIV [29]. Furthermore, substantial cultural differences regarding fatigue in children have been reported, precluding generalization of previous study results to PHIV+ living in the western world [30, 31]. No evidence is out there reporting on fatigue in children or adolescents who acquired HIV perinatally and are living in the western world.

Considering the high prevalence of fatigue in CCD and PLWH and the existing risk factors for developing fatigue in PHIV+, it is likely that fatigue is a prevalent symptom in PHIV+. This is important to know, as timely recognition and treatment of fatigue can decrease its burden and could prevent fatigue from continuing into adulthood [11, 32–34].

Therefore, the objective of this study is to investigate the extent of fatigue among PHIV+ children and adolescents. To reach this aim, we compared the symptoms of fatigue in PHIV+ to a control group matched for age, sex, region of birth, socioeconomic status (SES) and adoption status. To understand whether the extent and nature of fatigue symptoms in PHIV+ are similar to those of the general Dutch population or children with chronic disease, we used these two groups as a reference. In addition, we investigated the association between fatigue and HRQOL in PHIV+.

## Methods

### Participants and procedure

This cross-sectional study was part of the observational NOVICE study (neurological, cognitive, and visual performance in HIV-infected children), an open cohort study investigating the effects of perinatal HIV infection and the exposure of cART on neurological, cognitive and visual performances conducted at the Amsterdam University Medical Centers (AUMC) [2–4, 35–39]. Between February 2017 and July 2018, we recruited all PHIV+ children in the outpatient department of our hospital who were 8 years or older [38]. HIV-uninfected controls were recruited from similar communities and two government-licensed adoption organizations and were frequency matched to PHIV+ regarding age, sex, region of birth and socioeconomic status (SES) and adoption status [2, 3, 38]. Exclusion criteria for all participants were (non-HIV associated) chronic neurological diseases and severe psychiatric disorders [4]. All participants who were 12 years or older provided informed consent, together with parents or legal guardians of participants younger than 18 years old. The study protocol was approved by the ethics committee of the Amsterdam University Medical Center.

### Questionnaires

We measured fatigue using an online version of the Dutch self-report version of the Pediatric Quality of Life Inventory™ (PedsQL) Multidimensional Fatigue Scale (PedsQL MFS) for children aged 8–12 years and 13–18 years [40–43]. The PedsQL MFS was designed to measure fatigue in patients with acute and chronic health conditions as well as healthy populations. It includes 18 items, divided over three subscales: General Fatigue (six items, e.g., ‘I feel tired’; ‘I feel too tired to do things that I like to do’); Sleep/Rest Fatigue (six items, e.g., ‘I feel tired when I wake up in the morning’; ‘I rest a lot’); and Cognitive Fatigue (six items, e.g., ‘It is hard for me to keep my attention on things’; ‘It is hard for me to remember what people tell me’). The subscales are summarized into a Total Fatigue score. Items are rated on a 5-point Likert scale (0 = “never a problem”,1= “almost never a problem”,2 = “sometimes a problem”,3 = “often a problem”, and 4 = “nearly always a problem”) based on the preceding week. Items are reverse scored and linearly transformed to a 0–100 scale so that higher scale scores indicate fewer symptoms of fatigue. In this study, internal consistency reliability was α > 0.80 for Total Fatigue and the Sleep/Rest Fatigue and Cognitive Fatigue subscales, and α = 0.52 for the General Fatigue subscale [44].

We compared fatigue outcomes of PHIV+ with three groups: [1] a matched group of HIV-uninfected controls included in this study (as described above), [2] a reference group of children and adolescents from the general Dutch population and [3] a reference group of children and adolescents with chronic disease (CCD). Data of the reference group of the general population were previously collected at day care facilities and schools in the Netherlands [43]. This group consisted of 502 children, with a median age of 10.0 [5–13] years and 239 (47.6%) were male. Data of CCD were previously collected as part of the PROactive study [9] and included children with cystic fibrosis, autoimmune diseases, and children who completed treatment for cancer. CCD were 481 children with a median age of 11.0 (IQR 6–15) years, 202 (42%) were male.

We measured Health-Related Quality of Life (HRQOL) in PHIV+ and HIV-uninfected controls using an online version of the Dutch child self-report version of the generic Pediatric Quality of Life Inventory™ 4.0 (PedsQL™) [45, 46]. Higher scores indicate higher HRQOL. In this study, internal consistency reliability was α > 0.80 for the total score [44].

### Sociodemographic, adoption and HIV- and cART-related characteristics

For all participants we collected data on age, sex, region of birth, education level of (adoptive) parents and number of parents with a job. Educational level was scored according to the International Standard Classification of Education [ISCED] [47].

We performed laboratory testing of HIV-1 viral load (VL) and CD4+ T-cell count in all PHIV+ children. The Dutch HIV Monitoring Foundation provided additional data on HIV and cART (route of transmission, Centers for Disease Control and Prevention [CDC] category, nadir CD4+ T-cell z-score, zenith VL, age at cART initiation, HIV diagnosis to cART initiation).

We performed HIV-testing in the control group to confirm their HIV- uninfected status.

### Data analysis

We compared sociodemographic characteristics between PHIV+ participants and HIV-uninfected matched controls by using the unpaired T-Test or Mann-Whitney U test, and Fischer’s exact test for categorical data. We report mean ± standard deviation (SD) of normally distributed data; otherwise, we report median and interquartile range (IQR). We used regression analysis to explore differences in fatigue scores between PHIV+ and the three other groups. We used Shapiro-Wilk and Q-Q plots to check the assumption of normality. Since age and sex are associated with fatigue [28, 43], we adjusted the regression model for these variables. We present both adjusted and unadjusted mean differences, represented by unstandardized beta, and 95% confidence interval (CI). Within the PHIV+ group, we used linear regression to investigate the association between fatigue and HRQOL. We performed all statistical analyses in IBM SPSS Statistics (version 25). Since we considered these analyses to be exploratory we did not adjust for multiple comparisons.

## Results

### Participants

We examined 19 PHIV+ children from the outpatient department of our hospital for eligibility, of whom five (28%) declined consent and one (5%) did not meet the inclusion criteria. Thirteen (68%) provided consent to participate. We additionally included one PHIV+ child, receiving care in another treatment centre, whose adoptive parents approached us in response to the advertisements for healthy adopted controls. Finally, 14 PHIV+ and 14 HIV-uninfected matched controls participated in this study. All participants completed the questionnaires entirely. We present relevant sociodemographic characteristics of PHIV+ children and matched HIV- uninfected controls in Table 1. PHIV+ and HIV-uninfected matched controls did not significantly differ in age, sex, region of birth, SES or adoption status.

**Table 1:** Demographics of Perinatally Human Immunodeficiency Virus infected (PHIV+) children and HIV uninfected (HIV-) matched controls

Characteristics	n	PHIV+	n	HIV-	***P***^**a**^
**Number of participants**		14		14	
**Male sex**	14	5 (35.7%)	14	7 (50.0%)	0.710
**Age**, y, median (IQR)	14	10.2 (9.2–11.4)	14	10.0 (8.1–11.5)	0.571
**Region of birth**	14		14		0.174
Asia		1		0	
Sub-Sahara Africa		13		10	
Eastern Europe		0		2	
Central America		0		2	
**ISCED highest**, median (IQR)	13		14		0.248
0-2		2		0	
3-5		7		7	
6-8		4		7	
**Number of parents with a job**	13		13		0.202
1		6		2	
2		7		10	
**Adopted**	14	13 (93%)	14	12 (86%)	0.541
**Age at HIV diagnosis** (y), median (IQR)	14	2.10 (0.33–3.45)			
**Perinatal transmission**, n (%)	14	14 (100%)			
**CDC category**^a^, n (%) N/A	14				
N/A		12 (86%)			
B		1 (7%)			
C		1 (7%)			
**Nadir CD4+ T-cell z-score**^a^, median (IQR)	14	−0.76 (−1.35 to −0.13)			
**Zenith HIV VL** (copies/mL) ^a^, median (IQR)	14	107,492 (265–296,408)			
**cART**, n (%)	14	14 (100%)			
**Age at cART initiation** (y), median (IQR)	14	3.07 (1.06–5.41)			
**Duration of cART use** (y), mean (SD)	14	6.79 (2.56)			
**HIV diagnosis to cART initiation** (mo), median (IQR)	14	22.92 (4.00–59.38)			
**Undetectable HIV VL at assessment**, n (%)	14	14 (100%)			

Values are reported as n (%), unless otherwise stated. Undetectable is defined as HIV RNA < 40c/mL, and allowing viral blips. Values are reported as mean (SD), median (IQR) or n (%). *Abbreviations*: *n* Number, *PHIV+* Perinatally human immunodeficiency virus infected, *HIV−* HIV-uninfected controls, *y* Year, *IQR* Interquartile range, *ISCED* International Standard Classification of Education; Centers for Disease Control and Prevention, *N* Not symptomatic, A, mildly symptomatic; B, moderately symptomatic; C, severely symptomatic, *VL* Viral load, *mL* Milliliter, *cART* Combination antiretroviral therapy, *SD* Standard deviation, *mo* Month

^a^Since registration in the Netherlands

### Fatigue

**Fig. 1:**
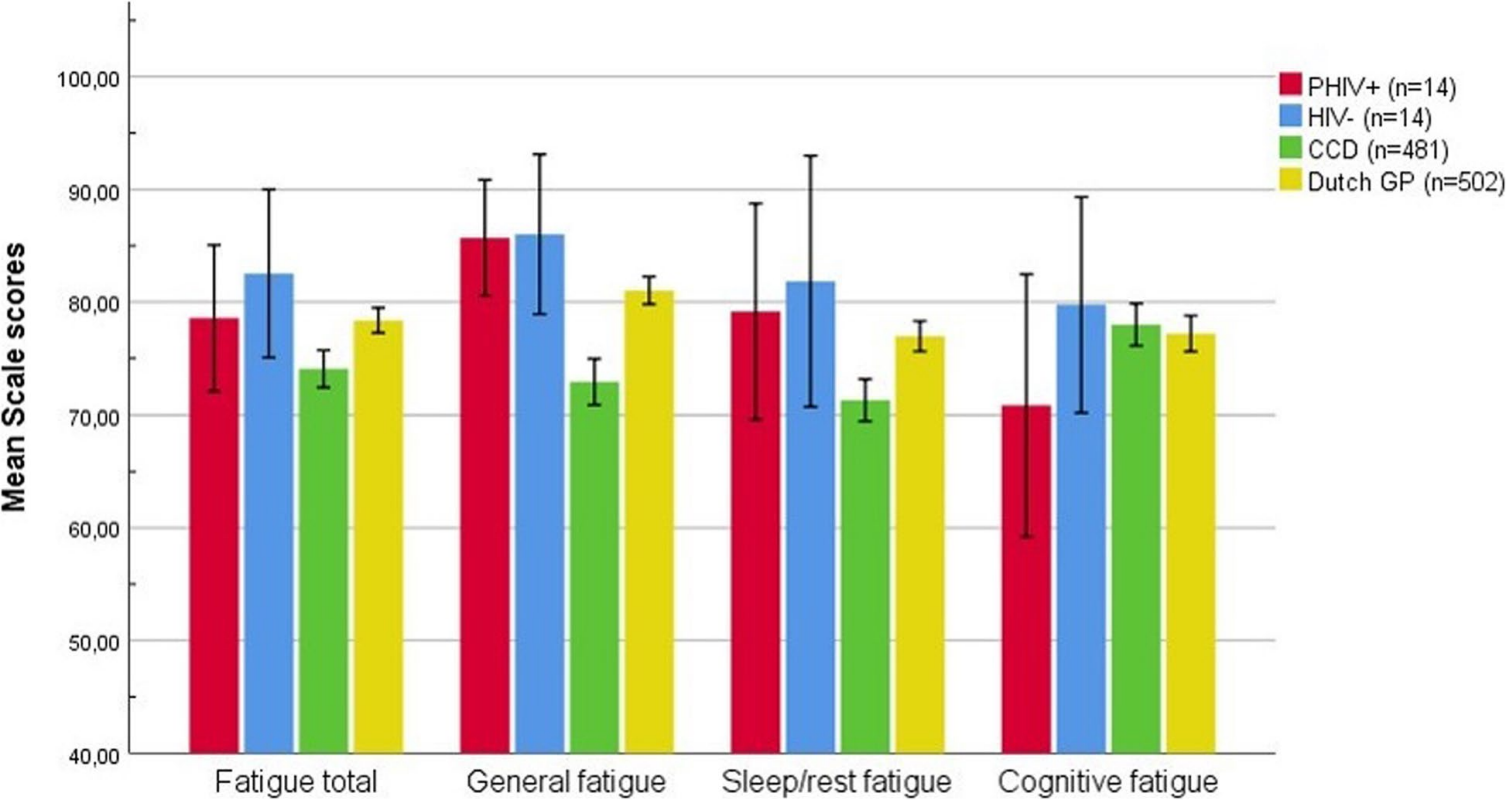
Mean Scale scores on PEDSQL MFS of PHIV+, HIV-, CCD and Dutch GP. Lower scores indicate more severely fatigued. Error bars represent 95% confidence intervals. Abbreviations: n, number; CCD, Children with chronic Disease; GP, General Population; PedsQL MFS; PedsQL multidimensional fatigue scale; PHIV+, perinatally human immunodeficiency virus infected; HIV−, HIV-uninfected matched controls

Table 2 and Fig. 1 present the PedsQL MFS scores of PHIV+ and HIV-uninfected matched controls for each separate scale. PHIV+ and HIV-uninfected matched controls did not significantly differ on any of the PedsQL MFS scores. Mean differences and 95% CI are presented in Table 2.

**Table 2:** PedsQL MFS Scores in PHIV+ and HIV-

	PHIV+ (*n* = 14)	HIV- (*n* = 14)	B^a^ (95% CI)	Adjusted B^ab^ (95% CI)
Fatigue total	78.57 ± 11.23	82.54 ± 12.93	−3.968 (− 12.9 to 5.0)	−4.520 (− 14.1 to 5.1)
General fatigue	85.71 ± 8.91	86.00 ± 12.30	−0.298 (− 8.3 to 7.7)	− 0.870 (− 9.5 to 7.7)
Sleep/rest fatigue	79.17 ± 16.59	81.85 ± 19.31	−2.679 (− 15.9 to 10.6)	−4.502 (− 15.4 to 6.4)
Cognitive fatigue	70.83 ± 20.15	79.76 ± 16.57	−8.929 (− 22.6 to 4.7)	−8.187 (− 22.9 to 6.5)

Fatigue scores are reported as mean ± SD. ^a^B represents mean difference between PHIV+ and the comparison group. ^b^Adjusted for age and sex. Lower scores indicate more fatigue; a negative B therefore means more fatigue in PHIV+ compared to HIV-. *Abbreviations*: *n* Number, *PedsQL MFS*, PedsQL multidimensional fatigue scale, *PHIV+* Perinatally human immunodeficiency virus infected, *HIV−* HIV-uninfected matched controls

Table 3 presents mean differences and 95% CI between PHIV+, CCD and the general Dutch population

**Table 3:** Mean differences in PedsQL MFS Scores between PHIV+ and HIV-, CCD and Dutch GP

	*CCD (n = 481)*		*Dutch GP (n = 502)*	
	B^a^ (95% CI)	Adjusted B^a^ (95% CI)	B^a^ (95% CI)	Adjusted B^ab^ (95% CI)
Fatigue total	4.501 (−5.2 to 14.2)	4.613 (−4.8 to 14.0)	0.195 (−6.6 to 7.0)	0.837 (−5.8 to 7.5)
General fatigue	**12.802 (0.8 to 24.8)**	**13.029 (1.3 to 24.8)**	4.685 (−2.7 to 12.0)	5.333 (−1.9 to 12.6)
Sleep/rest fatigue	7.857 (−3.1 to 18.8)	7.906 (−2.6 to 18.4)	2.203 (−6.0 to 10.4)	3.091 (−4.9 to 11.0)
Cognitive fatigue	−7.161 (−18.3 to 4.0)	− 7.109 (− 18.1 to 3.9)	− 6.366 (− 16.0 to 3.3)	−5.948 (− 15.6 to 3.7)

*B represents mean difference between PHIV+ and the comparison group. ^b^Adjusted for age and sex. Lower scores indicate more severely fatigued; a negative score therefore means more fatigue in PHIV+. Significant differences in bold. *Abbreviations*: *n* Number, *CCD* Children with chronic Disease, *GP* General Population, *PedsQL MFS* PedsQL multidimensional fatigue scale, *PHIV+* Perinatally human immunodeficiency virus infected, *HIV−* HIV-uninfected matched controls

Compared to CCD, PHIV+ children and adolescents scored less fatigued with respect to general fatigue (mean difference: 13.0 points; 95% CI 1.3 to 24.8). PHIV+ and CCD did not significantly differ on any of the other PedsQL MFS scores.

In comparison to the general Dutch population, PHIV+ children and adolescents did not score significantly different on any of the PedsQL MFS scales.

PHIV+ children scored relatively low on the cognitive fatigue scale in comparison to HIV-uninfected matched controls, CCD and the general Dutch population, although these differences did not reach significance.

### Health-related quality of life

PHIV+ and HIV-uninfected matched controls did not significantly differ on any of the HRQOL scores (see Table 1). Within the PHIV+ group, having a lower score on the total fatigue scale was significantly associated with a lower total HRQOL score (see Table 4). For each 1-point reduction in the PedsQL MFS score, the HRQOL was on average 0.73 points lower (95% CI 0.37 to 1.10). A lower score on total HRQOL was also associated with general fatigue (B 1.03; 95% CI 0.7 to 1.4) and cognitive fatigue (B 0.33; 95% CI 0.1 to 0.6), but not with sleep/rest fatigue.

**Table 4:** Association between PedsQL MFS and HRQOL in PHIV+

	HRQOL Total	
	B^a^	95% CI
Fatigue total	**0.733**	**0.4 to 1.1**
General fatigue	**1.028**	**0.7 to 1.4**
Sleep/rest fatigue	0.227	−0.1 to 0.6
Cognitive fatigue	**0.328**	**0.1 to 0.6**

^a^B represents the decrease in HRQOL score for each 1-point reduction in the PedsQL MFS score (indicating more fatigue). Significant differences in bold. PedsQL MFS; PedsQL multidimensional fatigue scale, HRQOL; Health-related quality of life

## Discussion

The results of this study suggest that PHIV+ children and adolescents do not experience more fatigue than healthy peers. In PHIV+ children, symptoms of fatigue are associated with HRQOL.

These results are not in line with earlier studies reporting a high prevalence of fatigue symptoms in children and adolescents living with HIV in South-Africa as well as in adults living with HIV in the Western world [10, 11, 19, 27, 28]. Neither do the symptoms of fatigue in PHIV+ look like those of another pediatric population with chronic disease, who generally experience more fatigue than the general population in all domains, except for cognitive fatigue [9]. On the contrary, in our study cognitive fatigue appeared to be the only domain that might be most affected in PHIV+ children and adolescents.

These findings of low levels of fatigue in PHIV+ might be because the majority of PHIV+ children and adolescents are treated optimally from an early age [48]. Consequently, most PHIV+ children have never (consciously) suffered from any HIV symptoms, in contrast to children with other chronic disease. Furthermore, the mean age of the population in this study is relatively low compared to the South-African study. While fatigue in PLWH is linked to psychosocial variables such as a depressed mood and cognitive functioning and the burden of these psychosocial variables increase as they grow up into adolescence and young adulthood, their experience of fatigue might increase too [4, 11, 18, 49–51]. In the healthy population, fatigue fluctuates naturally over the course of development and is more common in late adolescence [43, 52]. The same fluctuation of fatigue with age is found in adolescent PLWH [28]. We therefore suggest future studies should include older adolescents and young adults perinatally infected with HIV.

PHIV+ children and adolescents tended to report relatively more cognitive fatigue compared to all three reference groups. We previously reported a lower IQ and poorer cognitive profiles in this group, which might influence the reported cognitive fatigue [3, 4]. We therefore explored associations of cognitive fatigue with neuropsychological outcomes (IQ and Hotelling’s T). Indeed, these analysis showed a positive association between cognitive measures and cognitive fatigue (see Additional file 1, Table 2). Cognitive fatigue might arise as a result of increased cognitive demands, or the cognitive fatigue scale might be a proxy of perceived cognitive impairment more than fatigue specifically [12]. The association between fatigue and cognition is likely to be reciprocal, while it has been shown that experiencing cognitive fatigue can lead to a decrease in cognitive functioning, such as mental flexibility and planning [53–55]. This reciprocal relationship exposes the potential that treating fatigue could also have a positive effect on cognitive functioning. Treatment options such as cognitive behavioral therapy, exercise interventions and multifaceted treatment have shown promising results in other pediatric populations experiencing fatigue [56–58].

It is important to note some limitations to our study. Due to our inclusion criteria, participants in our study are relatively young, of high SES and more often adopted and born outside of the Netherlands compared to the total NOVICE cohort. PHIV+ participants did however not differ in terms of disease or treatment status. Along with the small sample size, this reduces the generalizability of the study results beyond PHIV+ with similar demographic characteristics. Furthermore, the small sample size prohibits us from drawing firm conclusions about the occurrence of fatigue in PHIV+. In a small sample there is a reduced chance of detecting a significant difference between PHIV+ and other groups when the true effect is small and variance is large [59]. The possibility of an existing effect can therefore not be dismissed. Simultaneously, it reduces the likelihood that a statistically significant result reflects a true effect. Therefore, we emphasize the importance of the suggestion of more cognitive fatigue in PHIV+ compared to all three groups, over any individually occurring significant outcomes. Small samples are also more at risk for outliers to severely skew outcomes. In our sample we found no outliers and therefore we included all data.

The age and sex distribution in the reference groups were not exactly the same as in the PHIV+ group [9, 43]. We attempted to compensate for this difference by adjusting our analyses for sex and age. We did not find any evidence that either influenced the results.

## Conclusions

In conclusion, optimally treated PHIV+ children and young adolescents living in the Netherlands do not experience more symptoms of fatigue then their healthy peers, although they do seem more likely to experience cognitive fatigue. These results need to be confirmed in studies with larger samples and in older populations of PHIV+ adolescents and young adults. Still, since fatigue is associated with health-related quality of life, we encourage health care practitioners to be attentive to symptoms of (cognitive) fatigue in PHIV+ children and adolescents.

## Supplementary Information

**Additional file 1.**

**Additional file 2.**

## Data Availability

The dataset supporting the conclusions of this article is included within Additional file 2 of this article. Previously collected data on reference groups study are available from MN, SN and RL, but restrictions apply to the availability of these data and are not publicly available.

## Abbreviations

ART: Antiretroviral therapy
cART: Combination antiretroviral therapy
CCD: Children with Chronic Disease
CDC: Centers for Disease Control and Prevention
CI: Confidence interval
HIV: Human immunodeficiency virus
HIV-: HIV-uninfected matched controls
HRQOL: Health related quality of life
IQR: Interquartile Range
ISCED: International Standard Classification of Education
PedsQL: Pediatric Quality of Life Inventory
PedsQL MFS: PedsQL Inventory Multidimensional Fatigue Scale
PHIV+: Perinatally HIV infected
PLWH: People living with HIV
SES: Socioeconomic status
SD: Standard deviation
VL: Viral Load
