# Supplementary material for: Fatigue in children and adolescents perinatally infected with human immunodeficiency virus: an observational study
Source: BMC Pediatr. 2021 Nov 20;21:519. doi: 10.1186/s12887-021-02977-6 (PMC8605599; doi:10.1186/s12887-021-02977-6)
Supplement: Supplementary file 1 — Additional file 1. [file 12887_2021_2977_MOESM1_ESM.docx]

# Additional file 1

**Additional table 1. HRQOL Scores in PHIV+ and HIV-.**

|  | PHIV+ (n=14) | HIV- (n=14) | B | 95% CI |
| --- | --- | --- | --- | --- |
| Physical functioning | 90.40±2.21 | 92.41±1.69 | -1.993 | -8.0 to 4.0 |
| Emotional functioning | 70.35±4.67 | 72.86±4.34 | -1.973 | -15.4 to 11.5 |
| Social functioning | 85.71±4.02 | 86.43±4.49 | -1.283 | -13.8 to 11.2 |
| School functioning | 75.36±3.72 | 80.36±4.18 | -6.150 | -17.8 to 5.5 |
| Total | 81.75±2.80 | 84.24±2.51 | -2.738 | -10.8 to 5.3 |
| Psychosocial functioning | 77.14±3.57 | 79.88±3.81 | -3.135 | -14.2 to 7.9 |

HRQOL scores are reported as mean±SD. B represents mean difference between PHIV and HIV-uninfected matched controls, adjusted for age and sex. Lower scores indicate a lower quality of life; a negative B therefore means lower HRQoL in PHIV+ compared to HIV-. Abbreviations: n, number; HRQOL, Health Related Quality of Life; PHIV+, perinatally human immunodeficiency virus infected; HIV−, HIV-uninfected matched controls.

**Additional table 2. Association between cognitive fatigue and cognitive test outcomes in PHIV+ and HIV-.**

|  | B* | 95% CI |
| --- | --- | --- |
| IQ* | -0.532 | **-0.1 to -1.0** |
| Hotelling’s T²* | -1.726 | **-0.6 to -2.9** |

*B represents the decrease in cognitive test outcome for each 1-point reduction in the PedsQL MFS score (indicating more fatigue). Hotelling’s T² is an outcome generated by multivariate normative comparison and reflects the degree of cognitive deviation of each participant compared to the control sample. Significant differences in bold. Abbreviations: IQ; Intelligence Quotient; PHIV+, perinatally human immunodeficiency virus infected; HIV−, HIV-uninfected matched controls.
